# Visual and anatomical outcomes of full-thickness macular hole surgery and associated factors in selected hospitals in Tanzania

**DOI:** 10.1371/journal.pone.0357451

**Published:** 2026-09-08

**Authors:** Heavenlight Estomihi Masuki, Jovin S. Sagale, Celina F. Mhina, Mustafa Yusufali, William Makupa, Milka Mafwiri

**Affiliations:** 1 Department of Ophthalmology, Muhimbili University of Health and Allied Sciences, Dar es Salaam, Tanzania; 2 Department of Ophthalmology, Comprehensive Community Based Rehabilitation in Tanzania (CCBRT) Hospital, Dar es Salaam, Tanzania; 3 Department of Ophthalmology, Kilimanjaro Christian Medical Centre, Moshi, Tanzania; Mayo Clinic Minnesota, UNITED STATES OF AMERICA

## Abstract

**Purpose:**

To determine the anatomical and visual outcomes of macular hole surgery and identify factors associated with surgical success among patients at Kilimanjaro Christian Medical Centre (KCMC) and Comprehensive Community-Based Rehabilitation Hospital (CCBRT), Tanzania.

**Methods:**

We conducted a retrospective cohort study between August and December 2023. Data on demographics, pre- and postoperative clinical characteristics, surgical details, and outcomes were extracted from medical records using a structured data collection tool. Analyses were performed using SPSS version 27. The Friedman test was used to compare median pre- and postoperative visual acuity (VA). Generalized estimating equations with logistic regression assessed factors associated with macular hole closure, while gamma regression modelled postoperative logMAR VA, accounting for repeated measures. Statistical significance was set at p < 0.05. Ethical approval was obtained from the Institutional Review Board, and patient data were handled confidentially.

**Results:**

A total of 101 eyes were analysed, of which all had idiopathic macular holes. Preoperatively, the median logMAR VA was 1.2(Snellen 6/96). At three months, median VA improved to 1.00(Snellen 6/60), representing a gain of 0.20 logMAR (approximately two ETDRS lines; p < 0.001). Anatomical closure was achieved in 70% of eyes. Multivariable analysis showed that good preoperative VA increased the odds of macula hole closure (aOR 19.77; 95% CI 1.20–25.3; p = 0.037). The use of the conventional ILM flap technique was associated with an increase in logMAR VA (aβ = 0.05, 95% CI 0.01–0.08, p = 0.012). Anatomical closure correlated with significant visual improvement (p < 0.001). Effect sizes for VA changes were modest, reflecting limited but meaningful functional gain.

**Conclusion:**

Over two-thirds of eyes achieved anatomical closure with modest visual improvement. These findings confirm the effectiveness of macular hole surgery in resource-limited settings. Expanding surgical capacity and applying evidence-based techniques could further improve outcomes. Longer-term follow-up with optical coherence tomography is recommended to optimize treatment strategies and patient counselling.

## Introduction

A macular hole (MH) is a vitreoretinal interface disease characterized by a retinal defect in the centre of the macula [1]. Macular holes can occur due to traumatic or idiopathic causes [2]. The macular hole can involve a few layers of the retina (lamellar macular hole) or the entire thickness of the retina (full-thickness macular hole) [3]. If left untreated, macular holes tend to enlarge, leading to loss of the central vision, which increases the risk of falls and accidents, thereby contributing to hospital morbidity [4–6].

Globally, the burden of visual impairment due to macular holes is 0.2–2.2 per 1000 population [4]. It accounts for a significant visual morbidity in the elderly population. Treatment of lamellar macula holes includes observation or ocriplasmin injection. Pars plana vitrectomy, ILM peeling and gas tamponade are widely accepted as the treatment of choice for full thickness macular holes [7]. Surgeries for macular holes were first done three decades ago in developed countries [6]. Studies on MH surgery outcomes report anatomical outcome (hole closure) range of 70% to 100% and a visual improvement of 10 more letters on a Snellen chart [6]. Factors associated with hole closure include the macula hole size, surgical technique and adjunctive procedure [8]. Factors associated with visual acuity included presenting visual acuity, and internal limiting flap technique employed.

Retinal surgery services, including macular hole repair, are available in only a handful of ophthalmology centres across Africa. The lack of robust data on surgical outcomes for macular holes in the region creates a critical evidence gap, providing the rationale for this study. In Tanzania, macular hole surgery was first introduced approximately two decades ago, and currently three tertiary eye units have the capacity to perform such procedures. However, the surgical outcomes and associated factors remain under-researched. Findings from this study will provide clinicians with insights to guide patient selection and better manage patients’ expectations following surgery. Therefore, this study aims to evaluate the visual and anatomical outcomes, along with their associated factors, of macular hole surgeries performed at selected hospitals in Tanzania.

## Materials and methods

A retrospective cohort study was conducted between 23/09/2023 to 15/12/2023 in two selected tertiary eye centres, namely Kilimanjaro Christian Medical Centre (KCMC) and CCBRT Hospital. KCMC, located in Moshi, Kilimanjaro, serves as the tertiary referral centre for Tanzania’s northern zone. Each year, approximately 520–780 vitreoretinal surgeries, including macular hole procedures, are performed by vitreoretinal surgeons as well as retina fellows. CCBRT Hospital, situated in Kinondoni Msasani, Dar es Salaam, provides a wide range of outpatient and surgical eye care services to over 90,000 patients annually. The hospital performs around 200 retinal surgeries each year and currently has one vitreoretinal surgeon.

The study included all patients aged 18 years and above who underwent macular hole surgery between October 2010 and December 2022 and completed at least three months of follow-up after surgery. Patients were excluded if their records had missing or incomplete information on pre- and post-operative visual acuity (VA) or optical coherence tomography data, if they underwent combined macular hole and retinal detachment surgery, or if they had coexisting proliferative diabetic retinopathy with macular hole. Patients with other causes of vision loss, such as optic neuropathy or age-related macular degeneration, were also excluded.

Data were collected using a structured questionnaire through a review of patients’ medical records. This included demographic information and preoperative clinical data such as presenting complaints, duration of symptoms, BCVA, MH type (idiopathic or traumatic), and OCT macular hole index (MHI). The MHI was calculated as the ratio between the hole height and the maximum basal diameter. Intraoperative details were also recorded, including the type of surgery (vitrectomy or phacovitrectomy), internal limiting membrane (ILM) peeling status (done/not done), dye used (brilliant blue or trypan blue), flap technique (conventional or inverted), tamponade agent used (silicone oil, Perfluoropropane [C3F8], or SF6), and any complications encountered.

Postoperative information included positioning instructions and surgical outcomes, namely anatomical and visual outcomes. The anatomical outcome of the MH was defined as ‘open’ when bare retinal pigment epithelium (RPE) remained exposed to the vitreous chamber, and ‘closed’ when there was complete retinal reconstitution without bare RPE, confirmed by OCT at the third postoperative month [9]. Visual outcome was defined as the best BCVA at the third postoperative month, measured using the Snellen chart and converted to LogMAR for analysis. Good visual acuity was defined as a BCVA of LogMAR ≤ 0.3, equivalent to a Snellen acuity of 6/12.

Data were analysed using SPSS version 27 software. Descriptive statistics summarized socio-demographic and clinical characteristics, with frequencies for categorical variables and medians with interquartile ranges (IQR) for numerical data. The Friedman test compared pre- and postoperative median logMAR visual acuity, while the Generalized Estimating Equation model was used for binary logistic regression (MH closure) and gamma regression (Visual outcome), accounting for repeated measures. Statistical significance was set at a p-value of 0.05 to identify significant risk factors for visual and anatomical outcomes.

This study was approved by the Institutional Review Board of Muhimbili University of Health and Allied Sciences. As only patients’ case notes were used for data collection, a waiver of informed consent was granted by the Institutional Review Board. The authors had access to information that could identify individual participants (such as names and hospital identification numbers) during data collection; however, only file numbers were recorded in the data collection tool, and all identifiers were removed prior to analysis. In addition, permission to conduct the study was obtained from the hospital directors of the selected hospitals

## Results

A total of 134 patients were recruited for the study. Thirty-seven patients were excluded from the analysis due to missing pre-operative visual acuity data (n = 8), absence of post-operative OCT data (n = 14), or having undergone combined macular hole and retinal detachment surgery (n = 8), or having a traumatic macular hole (n = 7). The final analysis included 101 eyes from 97 patients (93 uniocular and four binocular) with idiopathic macular holes. Two-thirds of patients (64, 66%) were aged over 60 years, while 33 (34%) were younger than 60 years. The median age was 63 years IQR (58–67), and there were slightly more females (53, 52.5%).

All eyes underwent pars plana vitrectomy with ILM peeling, using either gas or silicone oil tamponade. Of the 101 eyes which underwent macular hole surgery, 19 eyes required additional procedures: two eyes (10.5%) underwent combined macular hole and cataract surgery, two eyes (10.5%) underwent macular hole surgery with retinotomy, and 15 eyes (79%) underwent macular hole surgery with endolaser treatment for coexisting retinal breaks. Slightly more than half of the patients (53, 54.6%) had symptoms for more than six months, while 44 patients (45.4%) had symptoms for six months or less. The median symptom duration was six months (IQR 3–13 months)

The macula hole index was less than 0.5 in the majority of eyes (72; 71.3%). Perfluoropropane (C3F8) was the most common type of vitreous tamponade used (75, 74.3%). The ILM conventional flap was the most commonly used technique (88, 87.1%). Postoperative face-down positioning was performed by 66 patients (68%) (Table 1).

**Table 1:** Perioperative characteristics of the eyes with macular hole which underwent macular hole surgery, N = 101.

Variable	Frequency	
	n	%
**OCT macular hole index**		
Less than 0.5	72	71.3
More than 0.5	29	28.7
**Vitreous tamponade used**		
Silicon oil	2	2.0
C3F8	75	74.2
SF6	24	23.8
**ILM flap technique used**		
Conventional	88	87.1
Inverted	13	12.9
**ILM staining**		
Brilliant Blue	90	89.1
Trypan Blue	11	10.9
**Post op faces down position**		
Not documented	31	32.0
Yes	66	68.0

### Anatomical outcome

**Fig 1:**
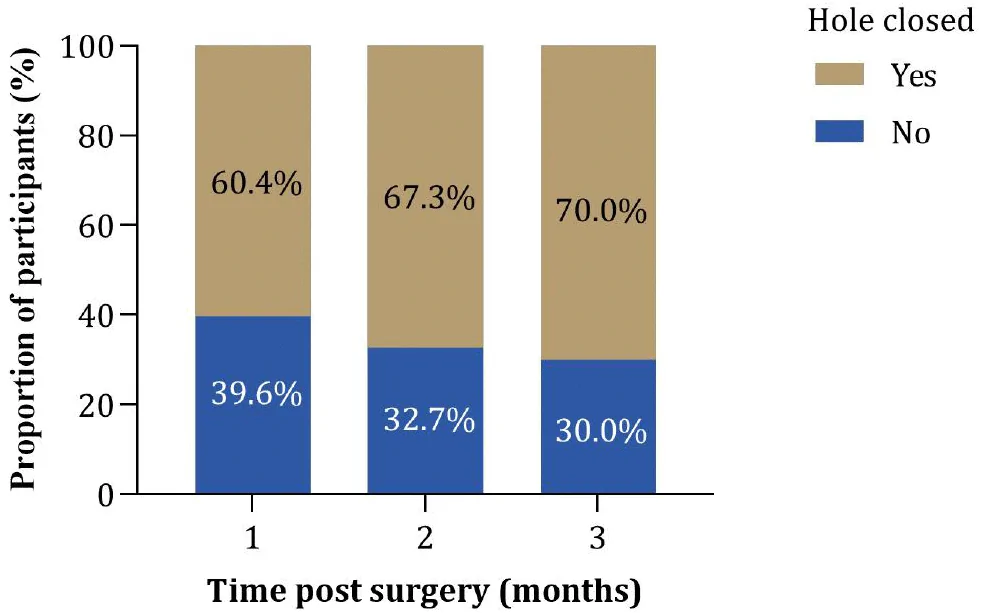
Proportion of eyes achieving macular hole closure at 1, 2, and 3 months postoperatively (N = 101), showing a steady increase over time.

Macula hole closure was achieved in 70% of operated eyes at the end of the 3-month follow-up (Fig 1).

### Visual outcome

More than one third (36, 35%) of eyes presented with VA of <3/60–1/60 before surgery Table 2. The median pre-operative VA was 1.2 log MAR (Snellen = 6/96)

**Table 2:** Preoperative BCVA distribution of patients’ eyes with macular hole, N = 101.

WHO category		N (%)
**Normal vision**	>6/12	0
**Mild visual impairment**	<6/12-6/18	7(07.0)
**Moderate visual impairment**	<6/18-6/60	34(33.7)
**Severe visual impairment**	<6/60-3/60	24(23.7)
**Blindness**	<3/60− 1/60	36(35.6)

**Fig 2:**
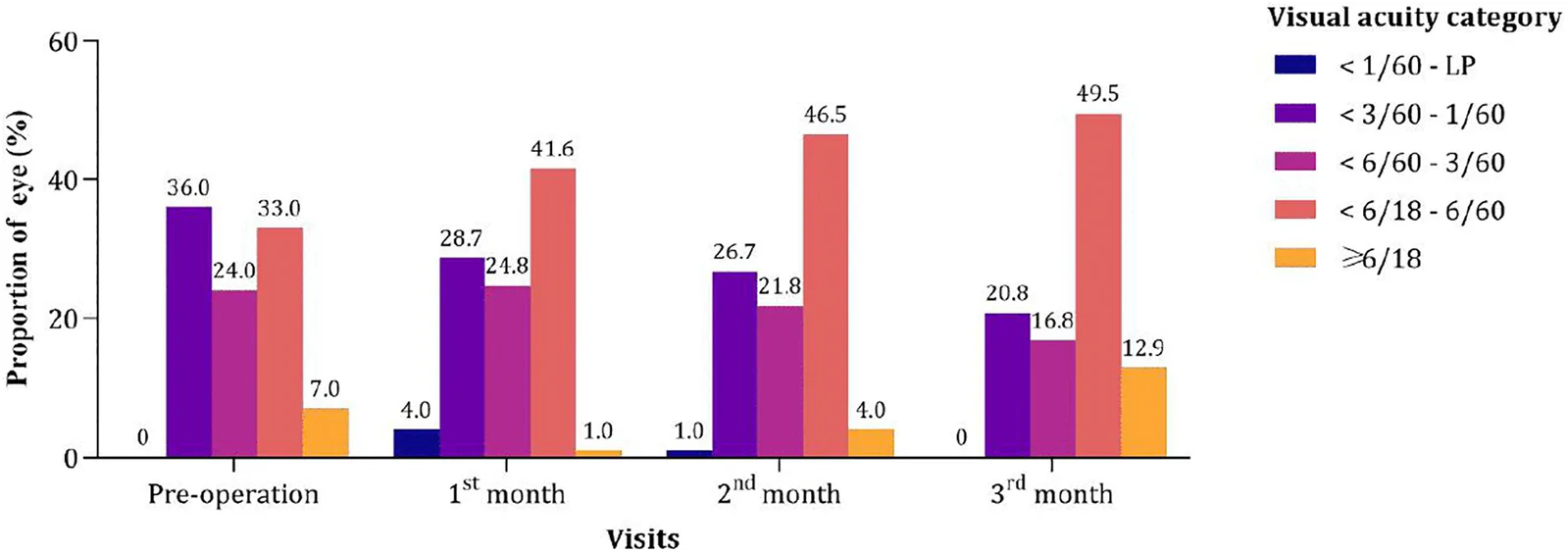
Preoperative and first 3 months postoperative best corrected visual acuity of patients’ eyes (N = 101).

Postoperatively, there was a marked reduction in the proportion of eyes with blindness (<3/60–1/60) from 36% preoperatively to 20.8% postoperatively and with severe (<6/60–3/60) visual impairment from 24.0% preoperatively to 16.8% at three months postoperatively. Conversely, the proportion of eyes with moderate impairment (<6/18–6/60) increased from 33% preoperatively to 49.5% at three months. The proportion of eyes achieving good vision (VA ≥ 6/18) also increased, consistent with overall visual improvement (Fig 2).

**Fig 3:**
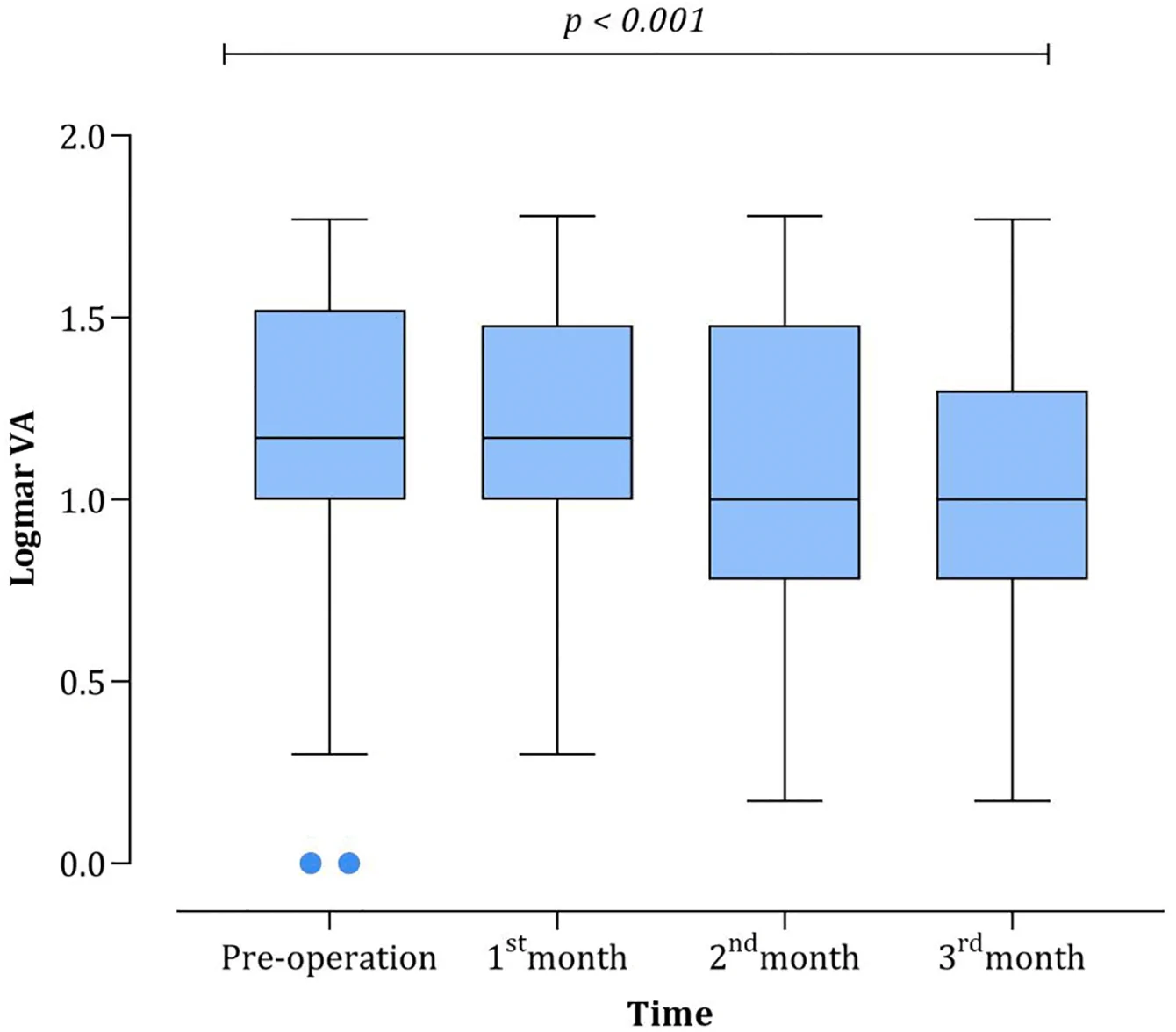
Median logMAR BCVA before and after surgery in eyes with macular hole (N = 101), showing significant postoperative improvement.

The median preoperative visual acuity (VA) was 1.2logMAR (Snellen equivalent 6/96). At three months postoperatively, the median VA improved to 1.00logMAR (Snellen 6/60), representing a median gain of 0.20 logMAR units. In practical terms, this improvement corresponds to approximately two ETDRS lines. The difference between preoperative and three-month postoperative BCVA was statistically significant (p < 0.001) (Fig 3).

Of the two eyes in which silicone oil was used as a vitreous tamponade, both achieved closure of the macular hole. However, due to the small number of eyes treated with silicone oil, meaningful comparison with gas tamponade could not be performed.

On multivariable analysis, good preoperative visual acuity was associated with significantly increased odds of macular hole closure (aOR 19.77; 95% CI 1.20–25.3; p = 0.037), Table 3.

**Table 3:** Univariable and multivariable logistic regression analysis of the factors associated with macular hole closure post-surgery, N = 99.

Variable	Category	Univariable analysis			Multivariable analysis		
		cOR	95% CI	p – value	aOR	95% CI	p–value
**Sex**	Female	1.20	0.82–1.77	0.354			
	Male	Ref					
**Duration**	>6 months	1.06	0.73–1.54	0.749			
	≤ 6 months	Ref					
**OCT MHI**	More than 0.5	1.18	0.75–1.86	0.474			
	Less than 0.5	Ref					
**Vitreous Tamponade used**	C3F8	1.35	0.94–1.94	0.109	1.41	0.93–2.13	0.104
	SF6	Ref					
**ILM flap technique**	Inverted	1.15	0.58–2.26	0.689			
	Conventional	Ref					
**ILM staining**	TB	1.33	0.56–3.15	0.514			
	BB	Ref					
**Pre-op VA (Logmar)**	Poor	Ref					
	Good	16.10	1.32–26.8	0.029	19.77	1.20–25.3	0.037

In multivariate regression analysis, use of conventional ILM flap technique was associated with a 0.05 (95% CI 0.01–0.08, p = 0.012) unit increase in LogMar VA. Other factors, including sex, duration of symptoms, ILM staining and macular hole index, were not significantly associated with visual outcome Table 4.

**Table 4:** Bivariable and multivariable regression analysis of the factors associated with Visual outcome N = 99.

Variable	Category	Univariable analysis			Multivariable analysis		
		cβ	95% CI	p – value	aβ	95% CI	p – value
Sex	Female	−0.04	−0.10–0.02	0.165	−0.05	−0.11–0.01	0.127
	Male	Ref					
Duration	>6 months	−0.03	−0.09–0.03	0.282			
	≤ 6 months	Ref					
OCT MHI	More than 0.5	0.03	−0.04–0.10	0.405			
	Less than 0.5	Ref					
Vitreous Tamponade used	SF6	−0.01	−0.07–0.04	0.623			
	C3F8	Ref					
ILM Flap technique	Conventional	0.05	−0.02–0.13	0.149	0.05	0.01–0.08	**0.012**
	Inverted	Ref					
ILM staining	BB	0.06	−0.03–0.14	0.197	0.02	−0.06–0.10	0.636
	TB	Ref					

Key: cβ: crude β-coefficient (slope), aβ: adjusted β-coefficient (slope), Ref: Reference.

**Fig 4:**
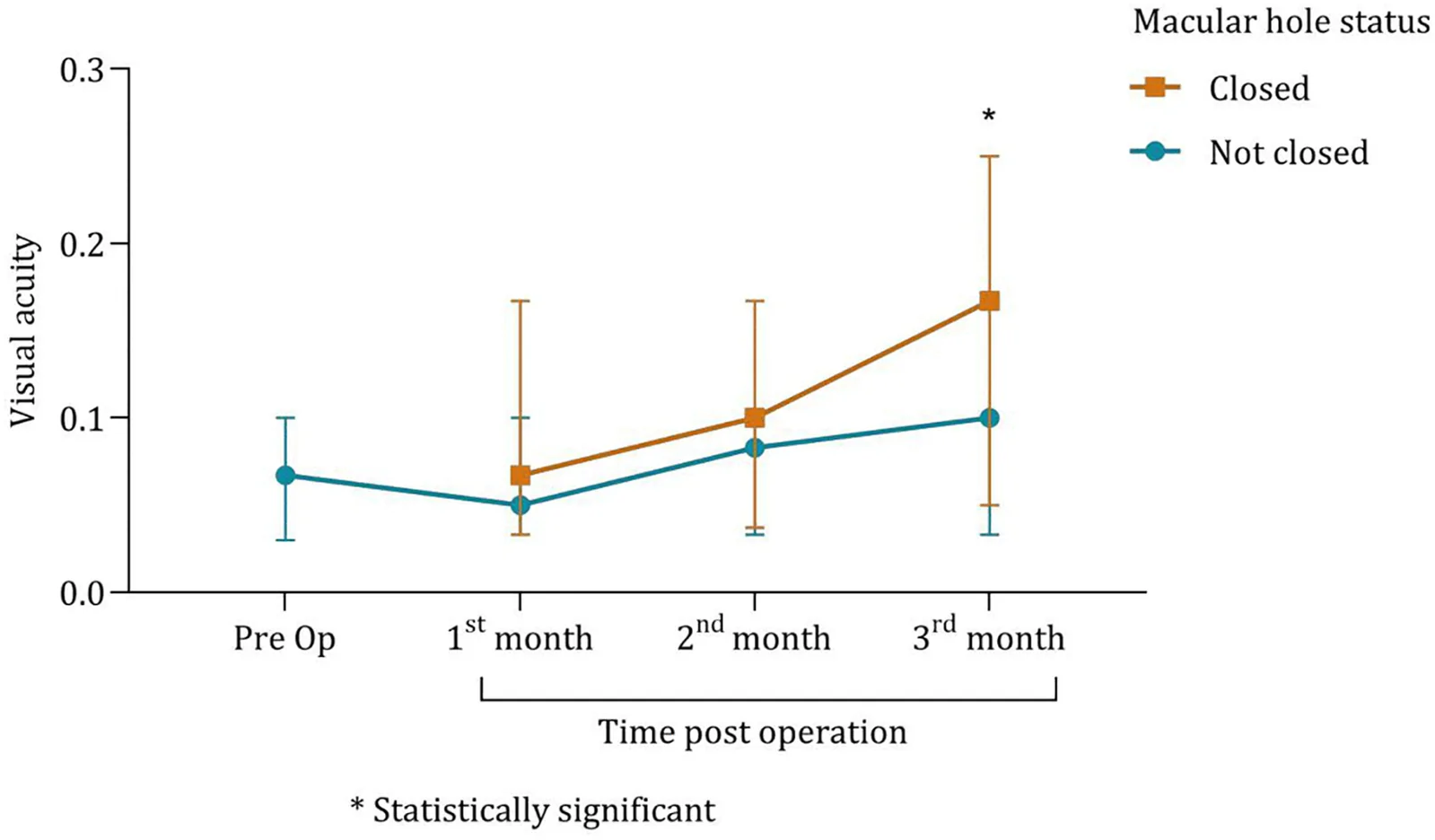
Visual acuity improvement over three months in eyes with and without macular hole closure.

Macular holes that achieved anatomical closure were also associated with a significant improvement in visual acuity, with the relationship reaching statistical significance at three months postoperatively (p < 0.001) (Fig 4).

## Discussion

Pars plana vitrectomy, ILM peeling, and gas tamponade are widely accepted as the treatment of choice for full-thickness macular holes. However, its effectiveness in achieving anatomical closure and visual restoration is unknown in our setting. This study sought to determine the outcomes of macular hole surgeries and to understand the associated factors to optimize patient outcomes in our setting. We reviewed the medical records of cases with full-thickness macular hole to determine closure rates and the visual outcomes.

In our study, over two-thirds of eyes achieved macular hole closure at three months postoperatively. This finding is similar to that reported by Chhablani et al. from Hyderabad, India, where 68.6% of eyes undergoing macular hole surgery achieved closure [10]. However, closure rates in our study were lower than those reported by Chandra et al. in London, UK, who found anatomical closure in 86.9% of eyes [11]. The differences in closure rates may be attributed to surgeon experience. Additionally, race and ethnicity were not routinely documented in our study and therefore could not be evaluated as potential factors associated with anatomical closure. This may be relevant given previous evidence suggesting ethnic variation in macular hole characteristics and surgical outcomes [11].

Of the two eyes in which silicone oil was used as a vitreous tamponade, both had persistent macular holes following initial surgery with gas tamponade and subsequently achieved macular hole closure after reoperation with silicone oil. Similar observations have been reported in other studies, which demonstrated good anatomical closure rates with silicone oil tamponade in cases of persistent or recurrent macular holes following vitrectomy with internal limiting membrane peeling and gas tamponade [12–15]. However, given the small number of eyes treated with silicone oil in our study, no meaningful comparison with gas tamponade could be made.

At three months postoperatively, the median logMAR VA in our study was 1.00 (Snellen equivalent 6/60), lower than that reported by Kaźmierczak et al. (0.30, 6/19) in Poland and Tirelli et al. (0.28, 6/11) in Italy [16,17]. We observed a median VA improvement of 0.20 logMAR (approximately two ETDRS lines), similar to Tirelli et al.’s findings [17], but less than the 0.30 logMAR improvement (three lines) reported by Steel et al. in the UK [18]. The relatively lower visual outcomes in our study may be partly explained by variability in surgeons’ experience, as some procedures were performed by less experienced surgeons. In addition, although longer symptom duration (observed in more than half of participants) and a macular hole index (MHI) <0.5 (seen in approximately two thirds of eyes) were not statistically significant in our study, these factors may have contributed to poorer vision, consistent with previous reports showing better outcomes in earlier-stage holes with shorter symptom duration [11,17,19].

In our study, the conventional flap technique was associated with a 0.05 increase in visual acuity compared to the inverted technique, indicating slightly poorer postoperative vision. This contrasts with the findings of Yamashita et al. (Japan), who reported no significant difference in visual outcomes between the two methods [20].

This study has several limitations. First, its retrospective design carries inherent drawbacks, including incomplete documentation and loss to follow-up. Key factors that could influence anatomical and visual outcomes, such as surgeon experience, assessment of near and colour vision, IOP changes, and postoperative positioning compliance, were excluded from the analysis due to inconsistent records. Similarly, important OCT parameters, including retinal cystic changes, vitreomacular interface status, macula hole size, and photoreceptor integrity, were unavailable, although these have been shown in other studies to correlate with surgical outcomes. The relatively short follow-up period further limited the ability to assess long-term visual recovery.

Future research should employ prospective study designs with standardized data collection, incorporate surgeon proficiency, and include more comprehensive OCT biomarkers and functional vision assessments. Longer follow-up will also be essential to better capture both anatomical closure and sustained visual improvement

## Conclusion

Over two-thirds of eyes achieved anatomical closure with modest visual improvement. These findings confirm the effectiveness of macular hole surgery in resource-limited settings. Expanding surgical capacity and applying evidence-based techniques could further improve outcomes. Longer-term follow-up with optical coherence tomography is recommended to optimize treatment strategies and patient counselling.

## Supporting information

S1 Data Minimal anonymised dataset underlying the findings reported in this study. (XLSX)
