# Supplementary material for: Visual and anatomical outcomes of full-thickness macular hole surgery and associated factors in selected hospitals in Tanzania
Source: PLoS One. 2026 Sep 8;21(9):e0357451. doi: 10.1371/journal.pone.0357451 (PMC13552745; doi:10.1371/journal.pone.0357451)
Supplement: Supplementary file 1 [file pone.0357451.s003.docx]

**Visual and anatomical outcomes of full-thickness macular hole surgery and associated factors in selected hospitals in Tanzania PONE-D-25-51893**

| JOURNAL REQUIREMENTS | AUTHOR'S RESPONSE |
| --- | --- |
| Please ensure that your manuscript meets PLOS ONE's style requirements, including those for file naming | Thank you  The Manuscript was formatted according to PLOS ONE standards, given from arrangement, headings, subheadings, tables, figures, fronts, and file naming |
| Please amend your authorship list in your manuscript file to include author Heavenlight Estomihi Masuki, [Jovin S Sagale](mailto:jovinsagale@gmail.com), [Celina F Mhina](mailto:c.mhina@yahoo.com). | Thank you  The Authorship list was amended in the manuscript to include Heavenlight Estomihi Masuki, [Jovin S Sagale](mailto:jovinsagale@gmail.com), [Celina F. Mhina](mailto:c.mhina@yahoo.com) |
| Please amend the manuscript submission data (via Edit Submission) to include author Heavenlight Masuki, Jovin Sagale, Celina Mhina. | Thank you. The Authorship list was amended in the submission data; now it includes Heavenlight Masuki, Jovin Sagale, and Celina Mhina. |
| [Please include captions for your Supporting Information files at the end of your manuscript, and update any in-text citations to match accordingly](http://track.editorialmanager.com/CL0/http:%2F%2Fjournals.plos.org%2Fplosone%2Fs%2Fsupporting-information./1/010f019c45b1a449-7025c061-600e-4857-bd93-c88a5b1239ee-000000/ogPRFMSJGXz1-_UtMKa-WbHbn4AjuQ3IOH32QL84niQ=249) | Thank you  The captions of the supporting information were added at the end of the manuscript |
| We note that there is identifying data in the Supporting Information file <Ethical clearance CCBRT.pdf, Ethical clearance KCMC.pdf, ethical clearance.docx | Thank you  The identifying data were anonymized, and re-uploaded |
| REVIWER 1COMMENTS |  |
| **Specific percentage be mentioned who achieved hole closure post-operatively** | Thank you.  The percentages were included 70% of the operated eyes attained hole closure |
| Authors have included 7 traumatic macular holes in their study group while majority being idiopathic. As visual prognosis and outcome get related to multiple factors, its better to keep traumatic holes away from the study group to have homogeneous inclusion and more significant data. | Thank you  The traumatic macular holes were removed, and analysis re done using the idiopathic macular holes only |
| 3) Authors have mentioned, silicone oil as a vitreous tamponade was associated with a higher success rate for macular hole closure compared to gas tamponade. Studies worldwide have mentioned silicone oil to be suitable for recurrent or complex macular holes, particularly those associated with retinal detachment and they usually end up having reduced anatomical success rates. Do authors have some more logical explanations in support of their finding? References : a) Cillino S, Cillino G, Ferraro LL et al. Treatment of persistently open macular holes with heavy silicone oil (Densiron 68) versus C2F6. A prospective randomized study. Retina. 2016;36(4):688–94. 74. b) Nowroozzadeh MH, Ashraf H, Zadmehr M, et al. Outcomes of light silicone oil tamponade for failed idiopathic macular hole surgery. J Ophthalmic Vis Res. 2018;13(2):130–7. 75 c) Lai JC, Stinnett SS, McCuen BW. Comparison of silicone oil versus gas tamponade in the treatment of idiopathic full-thickness macular hole. Ophthalmology. 2003;110(6):1170–4 | Thank you very much for this insightful comment.  In our study, silicone oil tamponade was used in a **very small number of cases (n = 2),** and both eyes achieved anatomical closure. We acknowledge that silicone oil is typically reserved for **complex or recurrent macular holes,** which are often associated with **lower closure rates** in the broader literature.  The observed higher success rate in our study should therefore be interpreted with caution. Given the **extremely small sample size**, this finding is highly susceptible to **random variation (chance effect)** and may not reflect a true clinical advantage of silicone oil over gas tamponades.  It is also possible that in our study surgeon-related factors contributed to the favorable outcomes observed, as silicone oil was used in persistent macular holes that were managed by more experienced surgeons compared to other cases. |
| REVIWER 2 COMMENTS |  |
| 1.The correlation of size of macular hole and anatomical closure and visual improvement will enhance value of the study. | Thank you for highlighting this important parameter. We agree that macular hole size is a key factor influencing surgical outcomes. However, we were unable to retrieve this information for the majority of patients due to inconsistent documentation. The data analyzed were extracted from patient clinical records, in which macular hole size was not routinely documented, and the only available OCT-derived parameter was the macular hole index. We acknowledge this as a limitation of our study and have clarified it in the revised manuscript. |
| The incidence of glaucoma in the study the effect of vision recovery in the eyes having glaucoma in the study | Thank you very much for this insightful comment. Unfortunately, due to inconsistent documentation of intraocular pressure (IOP) in the majority of patients, we were unable to assess the incidence of glaucoma or evaluate its impact on visual recovery. We acknowledge this as a limitation of our study and have clarified it in the revised manuscript. |
