# Supplementary material for: Visual and anatomical outcomes of full-thickness macular hole surgery and associated factors in selected hospitals in Tanzania
Source: PLoS One. 2026 Sep 8;21(9):e0357451. doi: 10.1371/journal.pone.0357451 (PMC13552745; doi:10.1371/journal.pone.0357451)
Supplement: Supplementary file 2 [file pone.0357451.s004.docx]

| Editors comments | Authors response |
| --- | --- |
| I share reviewer 1’s concern regarding the repeated claim that the success rate for silicon oil is higher than gas tamponade. Since only 2 of 101 eyes received a silicon oil tamponade, the higher success rate observed must be tempered with the fact that the silicon oil group is not sufficiently powered to compare to the gas tamponade group. For this reason I would strongly suggest that the authors change their statements to reflect the small sample size, and refrain from making forward statements about the higher success rate of silicon oil. If those eyes were first treated with a gas tamponade unsuccessfully, and then silicon oil, that would be a point worthy of discussion. | Thank you very much for this constructive comment  We agree that the silicone group included only two eyes, insufficiently powered to compare with the gas tamponade group. On reviewing the records, we found that both eyes had persistent macular holes following initial surgery with gas tamponade and subsequently achieved closure after reoperation with silicone oil. We have now included this finding in the Discussion. We also removed the two silicone oil cases from the comparative regression analysis and removed statements suggesting a higher success rate with silicone oil. The findings are now presented descriptively, and we state that no meaningful comparison between silicone oil and gas tamponade can be made because of the small number of cases (page 12) |
| I very strongly advise the authors to have a biostatistician review the statistical analysis of all the data. Again, I am concerned by the emphasis on the 2 eyes treated with silicon oil, which does not seem sufficiently powered to compare with gas tamponade causing me to question the other statistical data. In general, I always have a biostatistician check my own work, and I think it is a good practice. The biostatistician should be acknowledged in the manuscript | Thank you very much for this constructive comment. In response, the statistical analyses and interpretation of the findings were reviewed by a biostatistician. The biostatistician confirmed that, given the small number of eyes treated with silicone oil, a meaningful comparison between the silicone oil and gas tamponade groups was not appropriate. The remaining statistical analyses were considered appropriate. The regression analyses were therefore repeated after excluding the two eyes treated with silicone oil, and the revised results are presented in the manuscript. We have also acknowledged the biostatistician in the Acknowledgements section of the revised manuscript (page 14). |
| One wonders whether the lower success rate of macular hole repair in this study is due to patient compliance with positioning instructions? Do the authors have any thoughts on this? | Thank you very much for this constructive comment.  We agree that compliance with postoperative positioning instructions could potentially influence the anatomical closure rates observed in this study.  In our study, postoperative positioning was documented in 66 patients. However, adherence to the prescribed positioning could not be ascertained due to the study's retrospective design. Postoperative positioning documentation was unavailable for the remaining 31 patients. Therefore, we were unable to determine whether compliance with postoperative positioning contributed to the lower anatomical success rate observed in our study. We have acknowledged the inability to evaluate postoperative compliance as the limitation of the study. **page 13** |
| What is the racial/ethnic makeup of the cohort and was it uniform? If not, were there differences among groups? | Thank you very much for this constructive comment.  The study was conducted in Tanzania, and the majority of patients undergoing vitreoretinal surgery at the participating institutions are Black Africans. However, race and ethnicity were not routinely documented in the patients’ medical records. Therefore, we were unable to determine the racial/ethnic composition of the study cohort or assess differences in outcomes between racial or ethnic groups. |
