# Supplementary material for: Visual and anatomical outcomes of full-thickness macular hole surgery and associated factors in selected hospitals in Tanzania
Source: PLoS One. 2026 Sep 8;21(9):e0357451. doi: 10.1371/journal.pone.0357451 (PMC13552745; doi:10.1371/journal.pone.0357451)
Supplement: Peer review history

# Peer review: Decision Letter 0

**DOI:** 10.1371/journal.pone.0357451.r001

**Authors:** Kumar Saurabh

9 Feb 2026

Dear Dr. Masuki,

Thank you for submitting your manuscript to PLOS ONE. After careful consideration, we feel that it has merit but does not fully meet PLOS ONE’s publication criteria as it currently stands. Therefore, we invite you to submit a revised version of the manuscript that addresses the points raised during the review process.

Please submit your revised manuscript by Mar 26 2026 11:59PM. If you will need more time than this to complete your revisions, please reply to this message or contact the journal office at plosone@plos.org. When you're ready to submit your revision, log on to https://www.editorialmanager.com/pone/ and select the 'Submissions Needing Revision' folder to locate your manuscript file.

- A letter that responds to each point raised by the academic editor and reviewer(s). You should upload this letter as a separate file labeled 'Response to Reviewers'.
- A marked-up copy of your manuscript that highlights changes made to the original version. You should upload this as a separate file labeled 'Revised Manuscript with Track Changes'.
- An unmarked version of your revised paper without tracked changes. You should upload this as a separate file labeled 'Manuscript'.

If you would like to make changes to your financial disclosure, please include your updated statement in your cover letter. Guidelines for resubmitting your figure files are available below the reviewer comments at the end of this letter.

If applicable, we recommend that you deposit your laboratory protocols in protocols.io to enhance the reproducibility of your results. Protocols.io assigns your protocol its own identifier (DOI) so that it can be cited independently in the future. For instructions see: https://journals.plos.org/plosone/s/submission-guidelines#loc-laboratory-protocols. Additionally, PLOS ONE offers an option for publishing peer-reviewed Lab Protocol articles, which describe protocols hosted on protocols.io. Read more information on sharing protocols at https://plos.org/protocols?utm_medium=editorial-email&utm_source=authorletters&utm_campaign=protocols.

We look forward to receiving your revised manuscript.

Kind regards,

Kumar Saurabh

Academic Editor

PLOS One

Journal Requirements:

When submitting your revision, we need you to address these additional requirements.

1. Please ensure that your manuscript meets PLOS ONE's style requirements, including those for file naming. The PLOS ONE style templates can be found at

https://journals.plos.org/plosone/s/file?id=wjVg/PLOSOne_formatting_sample_main_body.pdf and https://journals.plos.org/plosone/s/file?id=ba62/PLOSOne_formatting_sample_title_authors_affiliations.pdf

2. Please amend your authorship list in your manuscript file to include author Heavenlight Estomihi Masuki, Jovin S Sagale, Celina F Mhina.

3. Please amend the manuscript submission data (via Edit Submission) to include author Heavenlight Masuki, Jovin Sagale, Celina Mhina.

4. Please include captions for your Supporting Information files at the end of your manuscript, and update any in-text citations to match accordingly. Please see our Supporting Information guidelines for more information: http://journals.plos.org/plosone/s/supporting-information.

5. We note that there is identifying data in the Supporting Information file <Ethical clearance CCBRT.pdf, Ethical clearance KCMC.pdf, ethical clearance.docx >. Due to the inclusion of these potentially identifying data, we have removed this file from your file inventory. Prior to sharing human research participant data, authors should consult with an ethics committee to ensure data are shared in accordance with participant consent and all applicable local laws.

Data sharing should never compromise participant privacy. It is therefore not appropriate to publicly share personally identifiable data on human research participants. The following are examples of data that should not be shared:

-Name, initials, physical address

-Ages more specific than whole numbers

-Internet protocol (IP) address

-Specific dates (birth dates, death dates, examination dates, etc.)

-Contact information such as phone number or email address

-Location data

-ID numbers that seem specific (long numbers, include initials, titled “Hospital ID”) rather than random (small numbers in numerical order)

Data that are not directly identifying may also be inappropriate to share, as in combination they can become identifying. For example, data collected from a small group of participants, vulnerable populations, or private groups should not be shared if they involve indirect identifiers (such as sex, ethnicity, location, etc.) that may risk the identification of study participants.

Additional guidance on preparing raw data for publication can be found in our Data Policy (https://journals.plos.org/plosone/s/data-availability#loc-human-research-participant-data-and-other-sensitive-data) and in the following article: http://www.bmj.com/content/340/bmj.c181.long.

Please remove or anonymize all personal information (<specific identifying information in file to be removed>), ensure that the data shared are in accordance with participant consent, and re-upload a fully anonymized data set. Please note that spreadsheet columns with personal information must be removed and not hidden as all hidden columns will appear in the published file.

6. Please remove all personal information, ensure that the data shared are in accordance with participant consent, and re-upload a fully anonymized data set.

Note: spreadsheet columns with personal information must be removed and not hidden as all hidden columns will appear in the published file.

Additional guidance on preparing raw data for publication can be found in our Data Policy (https://journals.plos.org/plosone/s/data-availability#loc-human-research-participant-data-and-other-sensitive-data) and in the following article: http://www.bmj.com/content/340/bmj.c181.long.

7. If the reviewer comments include a recommendation to cite specific previously published works, please review and evaluate these publications to determine whether they are relevant and should be cited. There is no requirement to cite these works unless the editor has indicated otherwise.

Additional Editor Comments:

Authors are invited to go through the reviewers comments and reply them suitably.

[Note: HTML markup is below. Please do not edit.]

Reviewers' comments:

Reviewer's Responses to Questions

**Comments to the Author**

1. Is the manuscript technically sound, and do the data support the conclusions?

Reviewer #1: Yes

Reviewer #2: Yes

**********

2. Has the statistical analysis been performed appropriately and rigorously?

Reviewer #1: Yes

Reviewer #2: Yes

**********

3. Have the authors made all data underlying the findings in their manuscript fully available?

The PLOS Data policy

Reviewer #1: Yes

Reviewer #2: Yes

**********

4. Is the manuscript presented in an intelligible fashion and written in standard English?

Reviewer #1: Yes

Reviewer #2: Yes

**********

Reviewer #1: I thank authors for their submission. Few queries from my side

1) Specific percentage be mentioned who achieved hole closure post-operatively

2) Authors have included 7 traumatic macular holes in their study group while majority being idiopathic. As visual prognosis and outcome gets related to multiple factors, its better to keep traumatic holes away from the study group to have homogeneous inclusion and more significant data.

3) Authors have mentioned, silicone oil as a vitreous tamponade was associated with a higher success rate for macular hole closure compared to gas tamponade. Studies worldwide have mentioned silicone oil to be suitable for recurrent or complex macular holes, particularly those associated with retinal detachment and they usually end up having reduced anatomical success rates. Do authors have some more logical explanations in support of their finding?

References :

a) Cillino S, Cillino G, Ferraro LL et al. Treatment of persistently open macular holes with heavy silicone oil (Densiron 68) versus C2F6. A prospective randomized study. Retina. 2016;36(4):688–94. 74.

b) Nowroozzadeh MH, Ashraf H, Zadmehr M, et al. Outcomes of light silicone oil tamponade for failed idiopathic macular hole surgery. J Ophthalmic Vis Res. 2018;13(2):130–7. 75

c) Lai JC, Stinnett SS, McCuen BW. Comparison of silicone oil versus gas tamponade in the treatment of idiopathic full-thickness macular hole. Ophthalmology. 2003;110(6):1170–4

Reviewer #2: 1.The correlation of size of macular hole and anatomical closure and visual improvement will enhance value of the study.

2. The incidence of glaucoma in the study . the effect of vision recovery in the eyes having glaucoma in the study.

**********

what does this mean?). If published, this will include your full peer review and any attached files.

If you choose “no”, your identity will remain anonymous but your review may still be made public.

**Do you want your identity to be public for this peer review?**  For information about this choice, including consent withdrawal, please see our Privacy Policy

Reviewer #1: No

Reviewer #2: No

**********

[NOTE: If reviewer comments were submitted as an attachment file, they will be attached to this email and accessible via the submission site. Please log into your account, locate the manuscript record, and check for the action link "View Attachments". If this link does not appear, there are no attachment files.]

To ensure your figures meet our technical requirements, please review our figure guidelines: https://journals.plos.org/plosone/s/figures

You may also use PLOS’s free figure tool, NAAS, to help you prepare publication quality figures: https://journals.plos.org/plosone/s/figures#loc-tools-for-figure-preparation.

NAAS will assess whether your figures meet our technical requirements by comparing each figure against our figure specifications.

# Author comment: Author response to Decision Letter 1

**DOI:** 10.1371/journal.pone.0357451.r002

1 May 2026

Reviewer 1 comments

1. Specific percentage be mentioned who achieved hole closure post-operatively

Authors response

Thank you.

The percentages were included 70% of the operated eyes attained hole closure

2. Authors have included 7 traumatic macular holes in their study group while majority being idiopathic. As visual prognosis and outcome get related to multiple factors, its better to keep traumatic holes away from the study group to have homogeneous inclusion and more significant data.

Authors response:Thank you

The traumatic macular holes were removed, and analysis re done using the idiopathic macular holes only

3.Authors have mentioned, silicone oil as a vitreous tamponade was associated with a higher success rate for macular hole closure compared to gas tamponade. Studies worldwide have mentioned silicone oil to be suitable for recurrent or complex macular holes, particularly those associated with retinal detachment and they usually end up having reduced anatomical success rates. Do authors have some more logical explanations in support of their finding?

Authors response: Thank you very much for this insightful comment.

In our study, silicone oil tamponade was used in a very small number of cases (n = 2), and both eyes achieved anatomical closure. We acknowledge that silicone oil is typically reserved for complex or recurrent macular holes, which are often associated with lower closure rates in the broader literature.

The observed higher success rate in our study should therefore be interpreted with caution. Given the extremely small sample size, this finding is highly susceptible to random variation (chance effect) and may not reflect a true clinical advantage of silicone oil over gas tamponades.

It is also possible that in our study surgeon-related factors contributed to the favorable outcomes observed, as silicone oil was used in persistent macular holes that were managed by more experienced surgeons compared to other cases.

REVIWER 2 COMMENTS

1.The correlation of size of macular hole and anatomical closure and visual improvement will enhance value of the study.

Authors response:Thank you for highlighting this important parameter. We agree that macular hole size is a key factor influencing surgical outcomes. However, we were unable to retrieve this information for the majority of patients due to inconsistent documentation. The data analyzed were extracted from patient clinical records, in which macular hole size was not routinely documented, and the only available OCT-derived parameter was the macular hole index. We acknowledge this as a limitation of our study and have clarified it in the revised manuscript.

2. The incidence of glaucoma in the study the effect of vision recovery in the eyes having glaucoma in the study

Authors response: Thank you very much for this insightful comment. Unfortunately, due to inconsistent documentation of intraocular pressure (IOP) in the majority of patients, we were unable to assess the incidence of glaucoma or evaluate its impact on visual recovery. We acknowledge this as a limitation of our study and have clarified it in the revised manuscript

Attachment Submitted filename: Response to Reviewers.docx

# Peer review: Decision Letter 1

**DOI:** 10.1371/journal.pone.0357451.r003

**Authors:** Alan Marmorstein

30 Jun 2026

Dear Dr. Masuki,

Thank you for submitting your manuscript to PLOS ONE. After careful consideration, we feel that it has merit but does not fully meet PLOS ONE’s publication criteria as it currently stands. Therefore, we invite you to submit a revised version of the manuscript that addresses the points raised during the review process.

Please submit your revised manuscript by Aug 14 2026 11:59PM. If you will need more time than this to complete your revisions, please reply to this message or contact the journal office at plosone@plos.org. When you're ready to submit your revision, log on to https://www.editorialmanager.com/pone/ and select the 'Submissions Needing Revision' folder to locate your manuscript file.

- A letter that responds to each point raised by the academic editor and reviewer(s). You should upload this letter as a separate file labeled 'Response to Reviewers'.
- A marked-up copy of your manuscript that highlights changes made to the original version. You should upload this as a separate file labeled 'Revised Manuscript with Track Changes'.
- An unmarked version of your revised paper without tracked changes. You should upload this as a separate file labeled 'Manuscript'.

If you would like to make changes to your financial disclosure, please include your updated statement in your cover letter. Guidelines for resubmitting your figure files are available below the reviewer comments at the end of this letter.

If applicable, we recommend that you deposit your laboratory protocols in protocols.io to enhance the reproducibility of your results. Protocols.io assigns your protocol its own identifier (DOI) so that it can be cited independently in the future. For instructions see: https://journals.plos.org/plosone/s/submission-guidelines#loc-laboratory-protocols. Additionally, PLOS ONE offers an option for publishing peer-reviewed Lab Protocol articles, which describe protocols hosted on protocols.io. Read more information on sharing protocols at https://plos.org/protocols?utm_medium=editorial-email&utm_source=authorletters&utm_campaign=protocols.

As the corresponding author, your ORCID iD is verified in the submission system and will appear in the published article. PLOS supports the use of ORCID, and we encourage all coauthors to register for an ORCID iD and use it as well. Please encourage your coauthors to verify their ORCID iD within the submission system before final acceptance, as unverified ORCID iDs will not appear in the published article. *Only*  the individual author can complete the verification step; PLOS staff *cannot*  verify ORCID iDs on behalf of authors.

We look forward to receiving your revised manuscript.

Kind regards,

Alan Marmorstein

Academic Editor

PLOS One

Journal Requirements:

If the reviewer comments include a recommendation to cite specific previously published works, please review and evaluate these publications to determine whether they are relevant and should be cited. There is no requirement to cite these works unless the editor has indicated otherwise.

Additional Editor Comments:

This is an interesting and informative retrospective analysis of macular hole outcomes in Tanzania. I have been asked to take over the review of the manuscript from the prior editor. Unfortunately I do not believe the authors have adequately responded to the prior review. In particular Reviewer 1 point 3. In addition, they were not able to provide the data on hole size or glaucoma requested by reviewer 2, though this is understandable in a retrospective study. I have several comments/questions that I think will significantly improve the manuscript:

1. I share reviewer 1’s concern regarding the repeated claim that the success rate for silicon oil is higher than gas tamponade. Since only 2 of 101 eyes received a silicon oil tamponade the higher success rate observed must be tempered with the fact that the silicon oil group is not sufficiently powered to compare to the gas tamponade group. For this reason I would strongly suggest that the authors change their statements to reflect the small sample size, and refrain from making forward statements about the higher success rate of silicon oil. If those eyes were first treated with a gas tamponade unsuccessfully, and then silicon oil, that would be a point worthy of discussion.

2. I very strongly advise the authors to have a biostatistician review the statistical analysis of all the data. Again, I am concerned by the emphasis on the 2 eyes treated with silicon oil, which does not seem sufficiently powered to compare with gas tamponade causing me to question the other statistical data. In general, I always have a biostatistician check my own work, and I think it is a good practice. The biostatistician should be acknowledged in the manuscript.

3. One wonders whether the lower success rate of macular hole repair in this study is due to patient compliance with positioning instructions? Do the authors have any thoughts on this?

4. What is the racial/ethnic makeup of the cohort and was it uniform? If not, were there differences among groups?

[Note: HTML markup is below. Please do not edit.]

Reviewers' comments:

Reviewer's Responses to Questions

**Comments to the Author**

Reviewer #2: All comments have been addressed

**********

2. Is the manuscript technically sound, and do the data support the conclusions?

Reviewer #2: Yes

**********

3. Has the statistical analysis been performed appropriately and rigorously?

Reviewer #2: Yes

**********

4. Have the authors made all data underlying the findings in their manuscript fully available?

The PLOS Data policy

Reviewer #2: Yes

**********

5. Is the manuscript presented in an intelligible fashion and written in standard English?

Reviewer #2: Yes

**********

Reviewer #2: (No Response)

**********

what does this mean?). If published, this will include your full peer review and any attached files.

If you choose “no”, your identity will remain anonymous but your review may still be made public.

**Do you want your identity to be public for this peer review?**  For information about this choice, including consent withdrawal, please see our Privacy Policy

Reviewer #2: No

**********

[NOTE: If reviewer comments were submitted as an attachment file, they will be attached to this email and accessible via the submission site. Please log into your account, locate the manuscript record, and check for the action link "View Attachments". If this link does not appear, there are no attachment files.]

To ensure your figures meet our technical requirements, please review our figure guidelines: https://journals.plos.org/plosone/s/figures

You may also use PLOS’s free figure tool, NAAS, to help you prepare publication quality figures: https://journals.plos.org/plosone/s/figures#loc-tools-for-figure-preparation.

NAAS will assess whether your figures meet our technical requirements by comparing each figure against our figure specifications.

# Author comment: Author response to Decision Letter 2

**DOI:** 10.1371/journal.pone.0357451.r004

12 Aug 2026

Editors comments Authors response

I share reviewer 1’s concern regarding the repeated claim that the success rate for silicon oil is higher than gas tamponade. Since only 2 of 101 eyes received a silicon oil tamponade, the higher success rate observed must be tempered with the fact that the silicon oil group is not sufficiently powered to compare to the gas tamponade group. For this reason I would strongly suggest that the authors change their statements to reflect the small sample size, and refrain from making forward statements about the higher success rate of silicon oil. If those eyes were first treated with a gas tamponade unsuccessfully, and then silicon oil, that would be a point worthy of discussion. Thank you very much for this constructive comment

We agree that the silicone group included only two eyes, insufficiently powered to compare with the gas tamponade group. On reviewing the records, we found that both eyes had persistent macular holes following initial surgery with gas tamponade and subsequently achieved closure after reoperation with silicone oil. We have now included this finding in the Discussion. We also removed the two silicone oil cases from the comparative regression analysis and removed statements suggesting a higher success rate with silicone oil. The findings are now presented descriptively, and we state that no meaningful comparison between silicone oil and gas tamponade can be made because of the small number of cases (page 12)

I very strongly advise the authors to have a biostatistician review the statistical analysis of all the data. Again, I am concerned by the emphasis on the 2 eyes treated with silicon oil, which does not seem sufficiently powered to compare with gas tamponade causing me to question the other statistical data. In general, I always have a biostatistician check my own work, and I think it is a good practice. The biostatistician should be acknowledged in the manuscript Thank you very much for this constructive comment. In response, the statistical analyses and interpretation of the findings were reviewed by a biostatistician. The biostatistician confirmed that, given the small number of eyes treated with silicone oil, a meaningful comparison between the silicone oil and gas tamponade groups was not appropriate. The remaining statistical analyses were considered appropriate. The regression analyses were therefore repeated after excluding the two eyes treated with silicone oil, and the revised results are presented in the manuscript. We have also acknowledged the biostatistician in the Acknowledgements section of the revised manuscript (page 14).

One wonders whether the lower success rate of macular hole repair in this study is due to patient compliance with positioning instructions? Do the authors have any thoughts on this? Thank you very much for this constructive comment.

We agree that compliance with postoperative positioning instructions could potentially influence the anatomical closure rates observed in this study.

In our study, postoperative positioning was documented in 66 patients. However, adherence to the prescribed positioning could not be ascertained due to the study's retrospective design. Postoperative positioning documentation was unavailable for the remaining 31 patients. Therefore, we were unable to determine whether compliance with postoperative positioning contributed to the lower anatomical success rate observed in our study. We have acknowledged the inability to evaluate postoperative compliance as the limitation of the study. page 13

What is the racial/ethnic makeup of the cohort and was it uniform? If not, were there differences among groups? Thank you very much for this constructive comment.

The study was conducted in Tanzania, and the majority of patients undergoing vitreoretinal surgery at the participating institutions are Black Africans. However, race and ethnicity were not routinely documented in the patients’ medical records. Therefore, we were unable to determine the racial/ethnic composition of the study cohort or assess differences in outcomes between racial or ethnic groups.

Attachment Submitted filename: Response to Reviwers.docx

# Editor report: Decision Letter 2

**DOI:** 10.1371/journal.pone.0357451.r005

**Authors:** Alan Marmorstein

18 Aug 2026

Visual and anatomical outcomes of full-thickness macular hole surgery and associated factors in selected hospitals in Tanzania

PONE-D-25-51893R2

Dear Dr. Masuki,

We’re pleased to inform you that your manuscript has been judged scientifically suitable for publication and will be formally accepted for publication once it meets all outstanding technical requirements.

Within one week, you’ll receive an e-mail detailing the required amendments. When these have been addressed, you’ll receive a formal acceptance letter and your manuscript will be scheduled for publication.

An invoice will be generated when your article is formally accepted. Please note, if your institution has a publishing partnership with PLOS and your article meets the relevant criteria, all or part of your publication costs will be covered. Please make sure your user information is up-to-date by logging into Editorial Manager at Editorial Manager® and clicking the ‘Update My Information' link at the top of the page. For questions related to billing, please contact billing support.

If your institution or institutions have a press office, please notify them about your upcoming paper to help maximize its impact. If they’ll be preparing press materials, please inform our press team as soon as possible -- no later than 48 hours after receiving the formal acceptance. Your manuscript will remain under strict press embargo until 2 pm Eastern Time on the date of publication. For more information, please contact onepress@plos.org.

Kind regards,

Alan Marmorstein

Academic Editor

PLOS One

Additional Editor Comments (optional):

Reviewers' comments:

# Editor report: Acceptance letter

**DOI:** 10.1371/journal.pone.0357451.r006

**Authors:** Alan Marmorstein

PONE-D-25-51893R2

PLOS One

Dear Dr. Masuki,

I'm pleased to inform you that your manuscript has been deemed suitable for publication in PLOS One. Congratulations! Your manuscript is now being handed over to our production team.

At this stage, our production department will prepare your paper for publication. This includes ensuring the following:

* All references, tables, and figures are properly cited

* All relevant supporting information is included in the manuscript submission,

* There are no issues that prevent the paper from being properly typeset

You will receive further instructions from the production team, including instructions on how to review your proof when it is ready. Please keep in mind that we are working through a large volume of accepted articles, so please give us a few days to review your paper and let you know the next and final steps.

Lastly, if your institution or institutions have a press office, please let them know about your upcoming paper now to help maximize its impact. If they'll be preparing press materials, please inform our press team within the next 48 hours. Your manuscript will remain under strict press embargo until 2 pm Eastern Time on the date of publication. For more information, please contact onepress@plos.org.

You will receive an invoice from PLOS for your publication fee after your manuscript has reached the completed accept phase. If you receive an email requesting payment before acceptance or for any other service, this may be a phishing scheme. Learn how to identify phishing emails and protect your accounts at https://explore.plos.org/phishing.

If we can help with anything else, please email us at customercare@plos.org.

Thank you for submitting your work to PLOS One and supporting open access.

Kind regards,

PLOS One Editorial Office Staff

on behalf of

Dr. Alan Marmorstein

Academic Editor

PLOS One
